# The use of electromyography and kinematic measurements of the lumbar spine during ergonomic intervention among workers of the production line of a foundry

**DOI:** 10.7717/peerj.13072

**Published:** 2022-03-18

**Authors:** Anna Błaszczyk, Małgorzata B. Ogurkowska

**Affiliations:** Department of Biomechanics, Poznan University of Physical Education, Poznan, Poland

**Keywords:** Biomechanics, Musculoskeletal disorders, Occupational ergonomics, Ergonomic intervention, Spine kinematics

## Abstract

**Purpose:**

Workers who perform repetitive movements are exposed to many risk factors leading to the occurrence of lumbar back pain. The aim of the research was to answer the question whether the ergonomic instruction conducted by a physiotherapist would change the tested range of motion of the segments of lumbar spine and the symmetry of the work of the torso and upper limbs muscles during work performed by foundry employees.

**Methods:**

The study included 12 foundry production line workers with musculoskeletal pain. The workstation was built in the laboratory that perfectly simulates work on the production line. The workers performed two activities from their daily work, i.e. transferring and moving a casting. They were then given ergonomic instruction by a physiotherapist and performed the assigned tasks again. During the activities, the electromyographic signal from five muscles was recorded in terms of symmetry of their work. The maximum angular ranges of the motor segments of the lumbar spine were measured using gyroscopes.

**Results:**

After the ergonomic instruction, the contralateral imbalance of muscle activity decreased statistically significantly during the first phase (*p* = 0.0004), second phase (*p* = 0.0002) and the third phase (*p* = 0.0069) of transferring the casting. While moving the casting , only erector spinae showed statistically significantly (*p* = 0.0131) more symmetrical work after the ergonomic instruction. During the transfer of the casting, statistically significantly lower values of the ranges of motion between the segments were obtained after carrying out the ergonomic instruction for the left (*p* = 0.0231) and right (*p* = 0.0032) lateral flexion. The ranges of movement between the segments differed statistically significantly for the flexion (*p* = 0.0117), extension (*p* = 0.0469) and left (*p* = 0.0031) and right lateral flexion movements (*p* = 0.0012).

**Conclusion:**

Conducting ergonomic instruction consisting in teaching the correct performance of a movement task reduced the contralateral imbalance of muscle activity and changes the ranges of movement of L1-S1 segments. The task of optimizing the load on the musculoskeletal system, including the lumbar spine, should be carried out by means of appropriate ergonomic instruction and multi-pronged measures, including analysis of the health of employees, their working environment and physical activity outside the workplace. Electromyography and measurements of the range of movement of the lumbar spine appear to be good tools for the evaluation of workplace ergonomics.

## Introduction

Musculoskeletal disorders (MSDs) are the most common condition in Europe (EU-OSHA). Risk factors such as performing repetitive movements, high production standards, carrying heavy objects, spine flexion and rotation are generally considered the main cause of MSDs among workers who perform hazardous manual handling tasks (Andersen et al., 2016; Andersen et al., 2017; Sterud & Tynes, 2013; Matsudaira et al., 2012). Workers performing manual lifting often excessively load the lower back, thereby putting themselves at risk of developing musculoskeletal disorders and spinal injuries (Jakobsen et al., 2018). Lower back pain (LBP) is the most common type of pain reported by workers in the automotive industry (Błaszczyk et al., 2020). Work-related musculoskeletal disorders also have direct negative economic consequences such as temporary disability/absence from work or early retirement (Andersen et al., 2012; Bevan et al., 2009).

Elimination of harmful factors prevents musculoskeletal disorders and has a positive impact on employees’ health (Macdonald & Oakman, 2013). This can be achieved through ergonomic interventions, which have a positive impact on: alleviating musculoskeletal pain, reducing the number of injuries, workers’ compensation claims and sickness absence days (Rivilis et al., 2008). Ergonomic interventions are defined as changes directed towards the improvement of a task, workstation, or work system from the ergonomic perspective (Rodríguez, 2018). A comprehensive approach to ergonomic interventions is the best solution to reduce the incidence of work-related MSDs (Dennerlein, 2017). Ergonomic interventions involve adjusting a workers’ environment, behavior, and other long-term educational approaches to treat and prevent further damage due to WMSD. A properly conducted ergonomic intervention reduces sickness absence related to upper limb pain (Shiri et al., 2011). Studies of this type are often conducted on office workers due to their static working position (Bazazan et al., 2019; Konarska et al., 2005); however, production line workers are also a large group of people affected by risk factors for work-related MSD. It should be noted that the employees of the foundry’s production line, due to the nature of their work, are subjected to dynamic loads. For the human musculoskeletal system, excessive static loads are unfavorable, but the dynamic ones are much worse (Kraemer, 2013). During a bending movement, there is a resultant load occurs from the torque of gravity of the trunk, upper limbs and head and in addition a torque of force due to the moment of inertia and angular acceleration of the trunk. It is this resultant torque of force that is the primary element in the pathobiomechanism of the sudden onset of lumbar spine pain during movement (Kraemer, 2013). Moreover, performing the same movement several hundred times a day is a risk factor for the occurrence of musculoskeletal disorders, however, performing the given movement repeatedly, and additionally not in accordance with the principles of ergonomics, will significantly aggravate the occurrence of ailments (Andersen et al., 2016; Andersen et al., 2017). The foundry’s production line workers move and put away heavy objects (10 kg), bending their spine up to 300 times a day. Additionally, they perform manual activities and torsional movements of the spine (Błaszczyk et al., 2020).

The biomechanical loads experienced in manual lifting and carrying loads depend on individual human preferences, in particular, the lifting style (Faber, Kingma & Dieën 2011; Hwang, Kim & Kim, 2009). Flexion-extension moments occurring at the L5-S1 level of the spine are significantly lower with the lower limb lifting technique than with the freestyle technique (Buseck et al., 1988). Additionally, this moment depends on the force arm, *i.e.* the distance at which the transferred load is held (Panjabi et al., 1989). The expressions ”holding the carried object as close to the body as possible, maintaining correct curvature of the spine while bending, engaging the lower limbs when bending” used during the ergonomic intervention have been defined as understandable to the study participants (Overton et al., 2016, Abdoli-Eramaki et al., 2019).

An ergonomic intervention may include physical therapy instruction, guidance on the use of tools, or redesign of workplaces (Rivilis et al., 2008; Shiri et al., 2011). Motion sensors such as accelero-, gonio- and inclinometers provide valuable information on the range of motion and inclination of body segments (Villumsen et al. 2015). Consequently, they can be used as a helpful tool during physiotherapeutic or ergonomic training. Surface electromyography can be used to monitor the degree of muscle activation before and after ergonomic instruction. Individuals with LBP show asymmetries or decreases in electromyographic signal (sEMG) compared to healthy individuals (Reger et al., 2006). A decrease in contralateral imbalances in muscle activity, therefore, may result in a decrease in lower back pain. When assessing the degree of muscle activation, it should be remembered that the use of surface electrodes may result in reading electrical potential from the area under study which does not have to equal the activation of the entire muscle (Vigotsky et al., 2017; Vieira & Botter, 2021).

Workers who perform repetitive movements are exposed to many risk factors leading to low back pain. Therefore, the aim of the research was to answer the question whether the ergonomic instruction conducted by a physiotherapist would change the tested range of motion of the segments of lumbar spine and the symmetry of the work of the torso and upper limbs muscles during work performed by foundry employees. Researchers hypothesize that following ergonomic instruction the angular range of motion in the lumbar spine will decrease, as will the difference in contralateral imbalances in electromyographic muscles activity.

## Materials & Methods

### Participants

The research group consisted of 12 men (Table 1) who were workers at two production line work stations in the foundry of automotive industry. The study was approved by the Bioethics Committee of the Poznan University of Medical Sciences (no. 561/18). All subjects expressed informed consent in writing to participate in the study. All procedures were conducted according to the 1964 Declaration of Helsinki. All subjects filled in a questionnaire (Błaszczyk et al., 2020) and reported musculoskeletal pain, especially in the lumbar spine (Table 2). The seniority of each subject at a particular position was at least 5 years (Table 1).

**Table 1:** Anthropometric characteristics of the studied cohort.

Variable	Mean ±*SD*
Age (years)	36.8 ± 8.3
Body height (cm)	180.1 ±5.4
Body mass (kg)	92.2 ± 11.4
Job seniority (years)	8.5 ± 3.8

**Table 2:** Lumbar segment where pathological changes occurred in the examined workers as diagnosed by a radiologist on the basis of a tomographic examination.

Spinal motion segment	Examined workers [%]
L1/L2	0
L2/L3	0
L3/L4	25
L4/L5	33
L5/S1	75

Workers who reported pain and in whom pathological changes in the lumbar spine were suspected underwent lumbar spine tomography evaluated by a radiologist.

**Figure 1:**
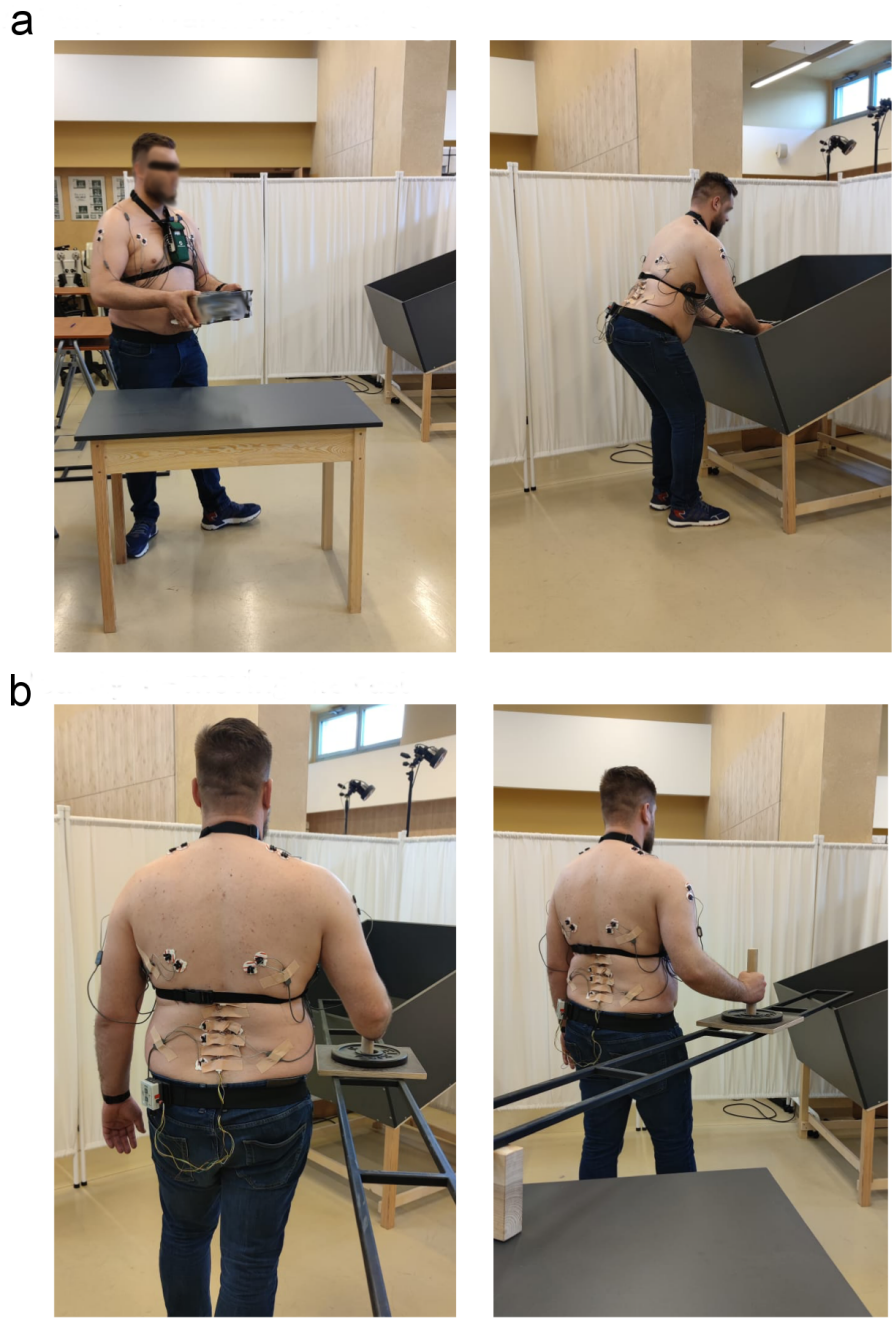
Work station for: (A) transferring the cast (activity I), (B) moving the cast (activity II).

Selected work stations required the workers to perform repetitive motion sequences (about 350/day). The workers carry heavy objects (10–12 kg), rotate and bend the spine while holding the load in their hands and also move the object using a special handle. Two selected work stations were reproduced under laboratory conditions (Fig. 1). It was not possible to carry out measurements in an industrial plant on the production line due to the presence of an electromagnetic field that interfered with the signal transmission of EMG measuring devices.

The work at the first work station consisted in transferring the original casting weighing 10 kg from the table into a box (Fig. 1A). It consisted of three parts, namely lifting the casting from the table at a height of 0.8 m, carrying it towards the box and then placing it in the box, the bottom of which was 0.5 m above the floor.

At the second work station, the men moved a weight, which imitated the carrying device, over a distance of 3 m (Fig. 1B). The force which had to be applied to move the item was measured with a dynamometer at the original work station and reproduced in the laboratory. The handle for moving the weight was at a height of 1.20 m.

### Electromyography

The surface EMG signal was recorded using the Telemyo 2400T G2 device (Noraxon, USA). Electrodes (Ag/AgCl SORIMEX, Poland, diameter: 1 cm) were placed on previously prepared skin (cleaned, shaved, degreased with alcohol) according to SENIAM guidelines ( Cram, Kasman & Holtz, 1998; Hermens et al., 2000; Kasman et al., 1998). Signals from the following 5 muscles were recorded bilaterally: erector spinae (ES), latissimus dorsi (LD), lower trapezius (LT), middle deltoideus (MD) and pectoralis major (PM). The examined muscles were identified in a pilot study based on a survey (Błaszczyk et al. 2020). The muscles of the back and the shoulder girdle were selected as the areas of the body which were described by workers as the most painful. For the EMG signal, 1,000 Hz sampling and filtering using a bandwidth of 10–500 Hz were applied (Merletti, 1999). The reference electrode was placed on the posterior superior iliac spine.

MyoResearch XP Master Edition software (Noraxon, USA) was used to process the signal. Artifacts were removed from the raw signal which then underwent full wave rectification and smoothing with the use of a root mean square (RMS) algorithm with a 50 ms window to form an amplitude estimation (Clancy, Morin & Merletti, 2002). Subsequently, the signal was normalised to the maximum voluntary contraction (% MVC) and the highest averaged EMG signal from the middle 1 second was used for analysis. Isometric MVC for all the muscles was determined in static conditions (Burden, 2010; Burden, Trew & Baltzopoulos, 2003; DeLuca 1997; Andreia & SousaJoao Manuel, 2012). The subject pressed with the corresponding body part with maximum force against a resistance shaft three times, maintaining maximum contraction for 3 seconds.

### Gyroscopes

The ZRP-3D6-BC (purchased from JBA Zb. Staniak) system was used to measure the spinal kinematics. The set was equipped with a recorder with six triaxial gyroscopes.

The test consisted in measuring the angular velocity and the angles traced by the individual motor segments of the vertebrae using the above mentioned system of triaxial sensors attached to the spinous processes of the five lumbar vertebrae and the sacral bone (L1-S1). The producer of the gyro systems in the technical specification gives a maximum linearity error of less than 1%. However, the accuracy of the measurement and calculation of the angle with this gyroscope is + - 1 degree. The measurement results were transmitted on-line during movement, directly to a computer *via* Bluetooth.

Using the CPG1v0 system software, the measured triaxial angular velocities were analysed to determine the maximum ranges of motion of the lumbar spine motor segments, *i.e.* L1/L2, L2/L3, L3/L4, L4/L5, L5/S1 in the sagittal and frontal plane during the two tested activities.

### Experimental protocol

The workers were given unlimited time to familiarise themselves with the test stand. They were then asked to transfer the casting from the table to the box on stand no. I (Fig. 1A) three times in the same way they do each day at work. Next, each worker received the same individual ergonomic instruction from one physiotherapist. The instruction consisted in communicating three pieces of information: holding the carried object as close to the body as possible, maintaining correct curvature of the spine while bending, engaging the lower limbs when bending. The worker performed the activity several times, introducing changes to the movement according to the instruction and following the physiotherapist’s comments. When, in the opinion of the instructor, he was able to perform a particular activity correctly, *i.e.* in compliance with the introduced ergonomic principles, he transferred the object three more times. The activity of transferring the casting was then divided into three phases using video recording (with the use of MyoResearch XP Master Edition):

-first phase of the movement –lifting the casting;

-second phase of the movement –carrying the casting;

-third phase of the movement –putting the casting down.

At stand no. II (Fig. 1B), the worker was asked to move an element along the rail three times in his own way, as he does every day at work. Then, the worker was given ergonomic instructions, which consisted in: assuming an appropriate position before work, so as not to perform rotation which additionally loads the motor system, and maintaining correct curvature of the spine while moving the element. Then, the employee practised the new way of performing the task several times, and when he could perform it as he was taught by physiotherapist (in compliance with the introduced ergonomic principles), three movement attempts were recorded. The EMG signal and angular values recorded with the ZRP-3D6-BC system from the three attempts were averaged for each of the two activities.

### Statistical calculations

Statistical calculations were performed using TIBCO Software Inc. (2017) Statistica (data analysis software system), version 13. For all electromyographic and kinematic variables, the reliability of the three measurements was calculated using the interclass correlation coefficient (ICC3,k) (95% confidence interval). Normality of data distribution was verified using the Shapiro-Wilk test. Differences in contralateral imbalances in muscle activity (ES, LD, LT, MD, PM) in time (before and after performing ergonomic instruction) and the differences in the range of motion between segments of the spine (L1/L2, L2/L3, L3/L4, L4/L5, L5/S1) in time (before and after performing ergonomic instruction) were calculated using multivariate analysis of variance with repeated measures (ANOVA 5x2). In the case of statistically significant differences between segments or muscles in time, Tukey’s post-hoc test (HSD) were performed. The level of statistical significance was set at *p* ≤ 0.05.

## Results

In order to investigate the inter-day reliability of the electromyographic measurements of individual muscles and kinematic measurements of vertebral motion for the activities of moving and transferring objects, an interclass correlation coefficient ICC(3,k) with a two-way mixed effect of consistency of multiple raters of the measurements was calculated (Koo & Li, 2016, Brandt et al., 2017). The ICC test-retest reliability level is good or excellent, with the values ranging from 0.769 to 0.999 (*p* < 0.001).

### Emg analysis

**Figure 2:**
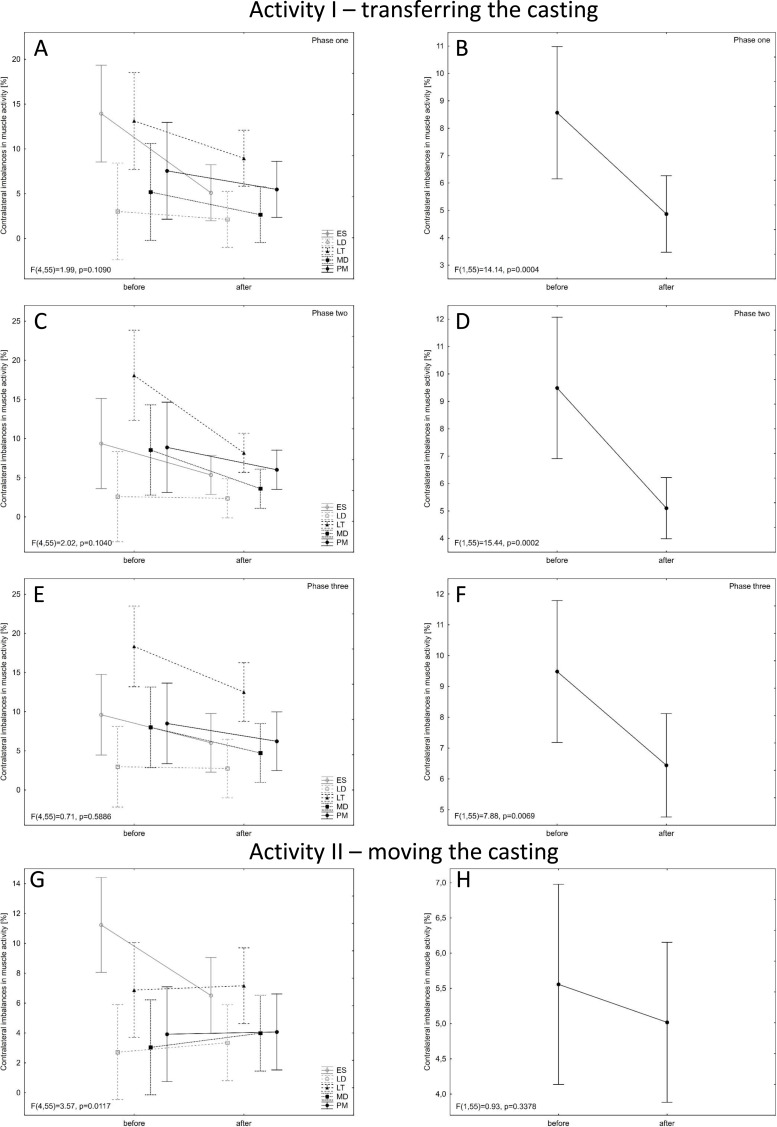
Contralateral imbalances in muscle activity before and after ergonomic instruction for the two activities (A, C, E, G for each muscle separately; B, D, F, G - general). Mean values with 95% confidence intervals are shown.

The objective of the EMG test was to check whether the difference in the electrical activity of a given muscle (right *versus* left side) was lower after the ergonomic instruction. It should be noted that a decrease in contralateral imbalances in muscle EMG was observed for all muscles during activity I (Figs. 2A–2F). After the ergonomic instruction, the contralateral imbalance of muscle activity decreased statistically significantly during the first phase (*p* = 0.0004) and in the second phase (*p* = 0.0002) and also in the third phase (*p* = 0.0069) (Figs. 2A–2F). In the case of Activity II, there was an interaction between contralateral imbalances of the muscles exam and the timing - before and after the instruction (5x2) (*p* = 0.0117). It should be noted here that during Activity II there are statistically significantly lower values of contralateral imbalances of ES activity after conducting ergonomic training, compared to the value before (*p* =0.0131) (Figs. 2G–2H).

### Gyroscopic analysis

The ZRP-3D6-BC system was used to assess the maximum range of motion between adjacent gyroscopes corresponding to consecutive motor segments of the lumbar spine. It was checked whether there was a statistically significant difference in this range before and after ergonomic instruction

### Activity I - transferring the casting

**Figure 3:**
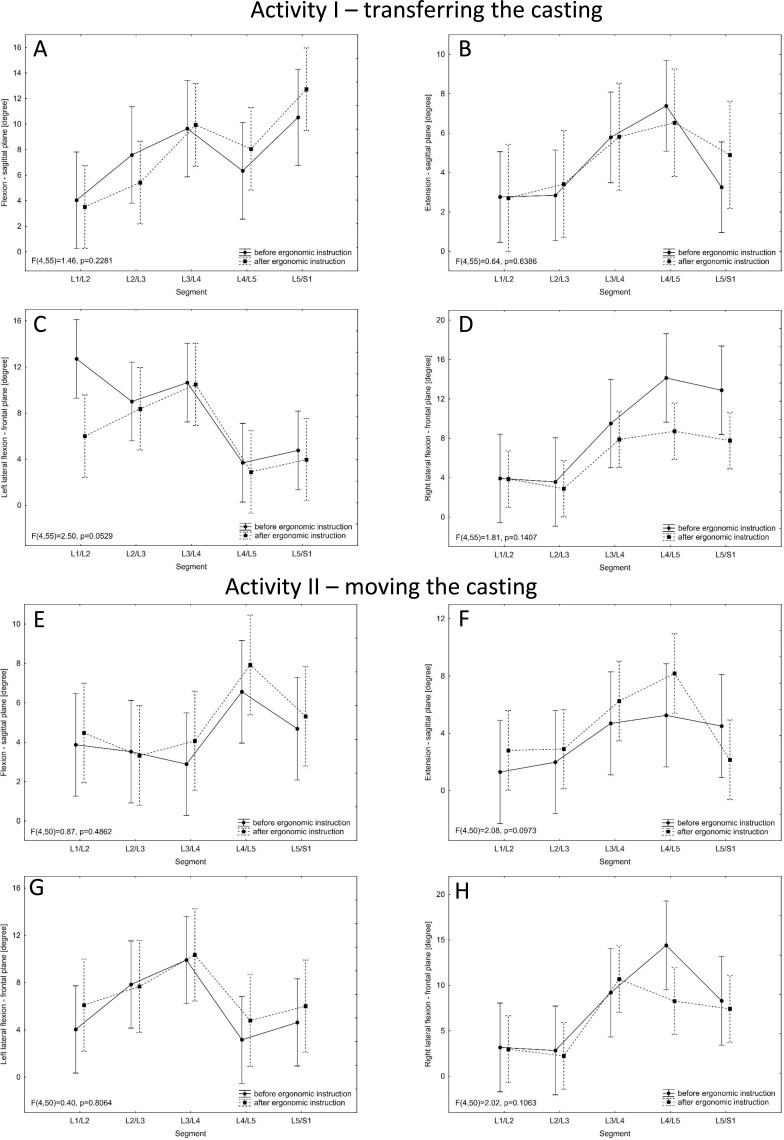
Values of the range of motion in a given spinal motor segment before and after the ergonomic instruction for the two activities. Mean values with 95% confidence intervals are shown.

In the case of the sagittal plane - the forward flexion movement of the spine, there were statistically significant differences between the range of motion of the segments (*p* = 0.0117), and more precisely, statistically significantly smaller values of movement in the L1/L2 segment than in the L5/S1 segment (*p* = 0.0087) (Fig. 3A). For the extension movement in the sagittal plane, there were statistically significant differences in the examined range of motion between the segments (*p* = 0.0469). There is a tendency towards significantly lower values in the L1/L2 segment than in the L4/L5 segment (*p* = 0.0710) (Fig. 3B).

In the case of the frontal plane—left lateral flexion of the spine, there are statistically significant differences between the range of motion in the segments of the lumbar spine (*p* = 0.0031) and more precisely significantly greater in L3 / L4 than in L4/L5 (*p* = 0.0108) and in L5/S1 (*p* = 0.0412) and also between L1/L2 and L4/L5 (higher in L1/L2, *p* = 0.0487). Statistically significant lower values of the ranges of movement between the segments were obtained after the training as compared to the values before (*p* = 0.0231) (Fig. 3C). For the frontal plane—right lateral flexion of the spine, there are statistically significant differences between the ranges of motion in the segments of the lumbar spine (*p* = 0.0012) and more precisely significantly lower in L2/L3 than in L4/L5 (*p* = 0.0069) and in L5/S1 (*p* = 0.0261) and also between L1/L2 and L4/L5 (lower in L1/L2, *p* = 0.0156). Statistically significant lower values of the ranges of movement between the segments were obtained after the ergonomic instruction as compared to the values before (*p* = 0.0032) (Fig. 3D).

### Activity II - moving the casting

The results obtained for the sagittal plane showed higher values of the range of motion occurred after than before the ergonomic training (flexion - *p* = 0.0181; extension - *p* = 0.1413) (Figs. 3E–3F).

When analyzing the results for the frontal plane - right lateral flexion, there were statistically significant differences between the range of motion between the segments (*p* = 0.0040), and more precisely significantly lower values in L2/L3 than in L4/L5 (*p* = 0.0160) and L5/S1 (*p* = 0.0272). After ergonomic instruction, lower values of the range of motion were obtained in the tested segments as compared to the values before (*p* = 0.1695) (Fig. 3H).

### Correlations

Correlations were sought between the delta (before *versus* after the instruction) of the EMG signal for the muscles in question and the delta (before *versus* after the instruction) of the range of motion in a particular motor segment. Two types of statistically significant correlations were determined in the case of activity I. For different pairs of parameters, both an increase and a decrease in the range of motion in a given spinal motor segment after the instruction correlated with a decrease in contralateral imbalances in muscle EMG (after the ergonomic instruction) (Table 3). In the case of activity II, similar statistically significant correlations were calculated and for one pair of variables a correlation was found where a decrease in the range of motion after the instruction correlated with an increase in contralateral imbalances in muscle work after the ergonomic instruction (Table 3).

**Table 3:** Summary of statistically significant (*p* < 0.05) correlations between the delta (before versus after the instruction) of the EMG signal for a given muscle and the delta (before versus after the instruction) of the range of motion in a particular segment.

Activity I					
Plane	Spinal motion segment	Muscle	Phase	*R* value	*p* value
SAGITTAL - FLEXION	L3/L4	LD	one	−0.6224^a^	0.0307
	L3/L4	MD	two	−0.5804^b^	0.0479
	L4/L5	PM	three	−0.6993^b^	0.0114
	L4/L5	ES	two	−0.6074^a^	0.0362
	L5/S1	LD	one	−0.5874^b^	0.0446
					
SAGITTAL- EXTENSION	L1/L2	PM	one	0.6853^b^	0.0139
	L2/L3	ES	one	−0.5771^a^	0.0495
					
FRONTAL - RIGHT LATERAL FLEXION	L1/L2	PM	two	−0.6014^b^	0.0386
	L2/L3	MD	three	0.5874^b^	0.0446
	L3/L4	LD	one	−0.6643^a^	0.0185
	L4/L5	MD	three	−0.8042^b^	0.0016
	L5/S1	PM	three	0.6783^b^	0.0153
Activity II					
SAGITTAL- EXTENSION	L5/S1	LD		−0.6182^b^	0.0426
FRONTAL - LEFT LATERAL FLEXION	L4/L5	ES		0.8455^b^	0.0010
FRONTAL - RIGHT LATERAL FLEXION	L5/S1	ES		−0.6636^b^	0.0260

**Notes.**

a Pearson’s correlation test.

b Spearman rank correlation test, *p* < 0.05 statistically significant value.

## Discussion

Comprehensive ergonomic evaluation of the two activities consisted primarily in the analysis of EMG signal from selected muscles and ranges of motion between segments of the lumbar spine before and after the ergonomic instruction. We were able to obtain the full clinical picture thanks to the previously conducted questionnaire study and analysis of tomography examinations of the lumbar spine.

After the ergonomic instruction, during the activity of transferring the casting, the contralateral imbalance of muscle activity decreased for all examined muscles (Figs. 2A–2F). The activity of lifting the casting was performed with both hands and the instruction had a positive effect on the movement technique. This was not observed in the case of moving the casting, which may be due to the fact that this movement was performed with one hand (asymmetric activity). Moreover, the occurrence of lumbar pain increases contralateral imbalances of muscle activity (Oddsson & Carlo, 2003). Also, imbalance or asymmetry of passive tissue could lead to asymmetry of muscular activation (Kim, Yoo & Choi, 2013). It is the changes within the passive tissue that are concomitant with the pathological changes found within intervertebral discs (Table 1). The significant decrease in ES electrical activity (Fig. 2G) is probably related to the change of the whole body posture resulting from the instruction. The employees adjusted the position of the trunk, but also engaged the lower limbs during the phase of putting down the casting or changed the grip on the casting while lifting it, at the same time paying attention to reducing the lever arm of gravity. Moreover, it should be emphasised that the weight of the casting before and after the instruction was the same, *i.e.* the spinal column in both cases was exposed to a pathobiomechanism which may cause musculoskeletal disorders (Ogurkowska & Kawałek, 2016). Therefore, optimising loads may be considered as the main task of the instruction. On the other hand, the change in body posture may have caused a redistribution of electrical ES activity. There are differences in amplitude depending on the place of EMG detection along the ES muscle (Serafino et al., 2021). Examined with the ZRP-3D6-BC system, the maximum ranges of motion between segments of the lumbar spine while transferring the casting prior to the instruction were similar to the reference values of mobility of individual segments (Panjabi et al., 1989; Adams, 2004). This means that the activity of putting down the casting was performed in a manner requiring full involvement of the musculoskeletal system and the full range of mobility of the lumbar spine in the sagittal and frontal planes. It should be emphasised that the ranges of motion of individual pairs of vertebrae examined in this study provide a much more precise picture of the pathobiomechanism of lumbar pain symptoms than an assessment of the entire L1-S1 segment. In the case of the sagittal plane, the range of motion increased together with the increase in the number of the lumbar vertebra, similarly to what Panjabi presented (1989). This was seen for both activity I and II. However, for the frontal plane, Panjabi determined that the middle segments of the lumbar spine had the greatest mobility (Panjabi et al., 1989). In the measured maximum ranges of motion for both activities, the highest values were also observed for the middle segments of this section (frontal plane).

Analysing the measured maximum ranges of motion before and after the instruction, for the sagittal plane, both an increase and a decrease in the angular ranges were observed. In response to the instruction, the subjects changed their movement pattern, which may have influenced the compensation phenomenon, *i.e.* in this case, a decrease in the range of movement in one segment may cause an increase in the range of movement in another segment. The pathobiomechanism of load transfer through the vertebral column means that the highest loads are carried by the lowermost segments (Kraemer, 2013; Ogurkowska & Błaszczyk, 2020). The force of gravity acting on the spine will consist of two components, *i.e.* the compression force and the shear force. The shear force has a negative effect and causes the protrusion of the intervertebral disc, which further leads to pain. More importantly, the shear force increases drastically due to movement, especially when leaning forward. In addition, this force is greater the lower the spine segments are located, which results from the angle of inclination of the intervertebral discs in relation to the transverse plane. It should be remembered that pathobiomechanical changes over time were studied. Performing repetitive movements in an incorrect way will lead to the formation of a hernia of the intervertebral disc and severe pain. Therefore, the study focused on the way of performing activities and the possibility of changing it through ergonomic instruction. (Adams et al., 2010). Increase in range of motion were observed at the L5/S1 level for spine flexion. Moreover, the presence of pathological changes visible in the tomographic image (the lower the motor segment, the more pathological changes–Table 2) may, on the one hand, be caused by excessive loading of the lower spinal segments and, on the other hand, disrupt the normal motor pattern and aggravate the phenomenon of compensation. The occurrence of degenerative changes within the intervertebral disc and the associated pain will disrupt the correct pattern of lifting/putting down a heavy object (Kraemer, 2013). A decrease in the range of motion for particular segments would indicate that the activity of putting down the casting is performed while maintaining the physiological curvature of the spine. Such position allows not to overload the intervertebral discs (equal distribution of pressure) and to prevent pathological changes (Kraemer, 2013). It should be remembered that all examined persons were diagnosed with pathological changes within the lumbar spine (Table 2). It may be associated with increased mobility within the spine segments due to improper functioning of the tissues stabilizing the spine (Ogurkowska & Kawałek, 2016). The decrease in the range of motion in the spine segments after the ergonomic training is a positive phenomenon. The lack of significant differences can be explained by the fact of increased mobility in segments associated with numerous degenerative changes. In addition, a single ergonomic instruction may not be a sufficient stimulus prompting changes in the developed/habitual movement pattern. Low effectiveness of a single instruction with simultaneous response to strength training at the workplace was observed in studies focusing on the assessment of musculoskeletal complaints (Sundstrup et al., 2020). For the frontal plane, however, a decrease in the ranges of motion between all segments was observed during the first activity. This change was the greatest for the L5/S1 segment (Figs. 3C–3D). This is the lowest segment of the lumbar spine, carrying the highest loads, which makes it the most vulnerable to the development of pathological changes (Ogurkowska & Błaszczyk, 2018). The decrease in contralateral imbalances of the activity of the muscles studied, in turn, correlated with both the decrease and increase in the ranges of motion of individual segments of the lumbar spine (Table 3). To achieve better results—reducing pain by improving posture and optimising loads during work—a multi-pronged intervention including ergonomic instruction, physical training and cognitive-behavioural training (CBT) would need to be introduced (Stevens et al., 2019). Early ergonomic intervention including assessment of the work environment, tools or devices operated, postures assumed while working, the need to carry heavy objects, pace of work and breaks, reduces sickness absence due to upper limb complaints or other musculoskeletal conditions (Shiri et al., 2011). This study has shown that this is not the case with a single instruction/discussion of the ergonomic situation in the workplace. Another way could be to extend the duration of the intervention up to several weeks. For workers with work-related neck-shoulder pain (WRNSP), the use of individualized motor control training and advice of ergonomic modifications at their workplaces resulted in reduced pain, increased range of motion of the cervical spine and reduced activity of bilateral upper trapezius muscle during active neck movements and functional tasks such as lifting things (Tsang et al., 2018).

### Limitation of the study

Due to the disturbances caused by the presence of the electromagnetic field in the foundry, it was not possible to conduct electromyographic and gyroscopic tests directly at the production line workstations. In addition, the EMG readings obtained depend on the accuracy of the electrode placement, and hence the experience of the tester. The workplace built in the laboratory was an accurate simulation of the production line located in the foundry, which was confirmed by the employees themselves. A limited number of employees took part in the study, as they were all people employed in these positions. The workplaces covered by the study were selected as those that burden the musculoskeletal system the most.

## Conclusions

Conducting ergonomic instruction consisting in teaching the correct performance of a movement task reduced the contralateral imbalance of muscle activity and changes the ranges of movement of L1-S1 segments. The task of optimizing the load on the musculoskeletal system, including the lumbar spine, should be carried out by means of appropriate ergonomic instruction and multi-pronged measures, including analysis of the health of employees, their working environment and physical activity outside the workplace. Electromyography and measurements of the range of movement of the lumbar spine appear to be good tools for the evaluation of workplace ergonomics.

## Supplemental Information

10.7717/peerj.13072/supp-1 Supplemental Information 1 Data from electromyographic and gyroscopic measurements collected before and after ergonomic training This collection was used to perform statistical calculations.

## Additional Information and Declarations

### Data Availability

The following information was supplied regarding data availability: The raw data are available in the Supplementary File.
